# Parvalbumin interneuron deficits in schizophrenia

**DOI:** 10.1016/j.euroneuro.2024.02.010

**Published:** 2024-05

**Authors:** Oscar Marín

**Affiliations:** a Centre for Developmental Neurobiology, Institute of Psychiatry, Psychology and Neuroscience, King's College London, London SE1 1UL, United Kingdom; b Medical Research Council Centre for Neurodevelopmental Disorders, King's College London, London SE1 1UL, United Kingdom

**Keywords:** cerebral cortex, interneuron, GABA, parvalbumin, fast-spiking, schizophrenia, post-mortem, EEG, MEG

## Abstract

Parvalbumin-expressing (PV+) interneurons represent one of the most abundant subclasses of cortical interneurons. Owing to their specific electrophysiological and synaptic properties, PV+ interneurons are essential for gating and pacing the activity of excitatory neurons. In particular, PV+ interneurons are critically involved in generating and maintaining cortical rhythms in the gamma frequency, which are essential for complex cognitive functions. Deficits in PV+ interneurons have been frequently reported in postmortem studies of schizophrenia patients, and alterations in gamma oscillations are a prominent electrophysiological feature of the disease. Here, I summarise the main features of PV+ interneurons and review clinical and preclinical studies linking the developmental dysfunction of cortical PV+ interneurons with the pathophysiology of schizophrenia.

## 1 Introduction

Schizophrenia is a complex and heterogeneous disorder generally characterised by recurrent psychosis, deterioration of social life, and cognitive dysfunction (Carpenter and Buchanan, 1994; Howes and Murray, 2014; Kahn et al., 2015). Schizophrenia symptoms are often classified into positive, negative, and cognitive categories, although they are by no means exclusive to the disorder. Positive symptoms are linked to recurrent episodes of psychosis driven by hallucinations and delusions and characterised by loss of contact with reality, disorganised speech, and agitated behaviour. Negative symptoms are often pervasive and include social withdrawal, affective flattening, anhedonia, and lack of initiative. Schizophrenia is also often associated with broad cognitive deficits impacting executive functions, including working memory, cognitive flexibility, planning, and metacognition.

Schizophrenia is diagnosed following the recurrence of psychotic episodes, generally during late adolescence, but the onset of the illness may precede the first psychotic episode by more than ten years. Indeed, cognitive development is compromised early in life in patients later diagnosed with schizophrenia (Kahn and Keefe, 2013), and differences have also been reported for critical neurodevelopmental milestones and associated behaviours (Hyde et al., 2008; Jääskeläinen et al., 2008; Sørensen et al., 2010). These observations are consistent with the prevailing hypothesis that schizophrenia is caused by changes in the typical trajectory of brain development (Murray and Lewis, 1987; Weinberger, 1987), which progressively lead to alterations in brain function and behaviour. Indeed, many of the genes that have been consistently associated with schizophrenia are expressed early in life and control critical aspects of brain development (Pocklington et al., 2015; Ripke et al., 2014; Singh et al., 2022; Trubetskoy et al., 2022), and many epidemiological risk factors are linked to early life events, such as obstetric complications and childhood adversity (Cannon et al., 2002; Stilo and Murray, 2010).

Ultimately, schizophrenia is the result of altered development trajectories that affect how the brain processes information and adapts to life experiences. However, identifying consistent pathophysiological changes in the brains of schizophrenia patients has proven difficult. Gross brain pathology is not a characteristic of schizophrenia. Instead, postmortem studies suggest alterations in synaptic connectivity and the functional state of specific neuronal and glial cell populations. Although postmortem studies are limited by a myriad of epiphenomena (e.g., treatment, chronicity, comorbidities), several observations have been replicated across multiple studies. For instance, multiple studies have found structural alterations in the dendrites of glutamatergic neurons across multiple regions of the cerebral cortex in schizophrenia patients, including diminished dendrite length and complexity and reduced spine density (Hu et al., 2015). Another highly replicated finding in the brain of schizophrenia patients is a reduction in the expression of one of the biosynthetic enzymes of GABA, glutamate acid decarboxylase 67 (GAD67), along with other GABAergic markers (Dienel and Lewis, 2019). Postmortem evidence of alterations in the dopaminergic system in patients with schizophrenia has been more inconsistent. However, data from positron emission tomography (PET) neuroimaging studies have repeatedly found increased striatal dopamine uptake in schizophrenia patients (Kesby et al., 2018; McCutcheon et al., 2019).

Positive symptoms in schizophrenia are generally associated with the presynaptic dysregulation of the dopaminergic modulation of striatal function, likely caused by abnormal activation of midbrain dopaminergic neurons (Howes et al., 2012; Lyon et al., 2011; Murray et al., 2008). Striatal dopaminergic dysfunction has been linked with psychosis-driven agitation and aberrant salience processing, but it is unclear how it may relate to the emergence of hallucinations. Abnormalities linked to negative symptoms may also be related to reward processing and emotional regulation. For example, ventral striatal responses to reward are consistently reduced in schizophrenia (Juckel et al., 2006), and deficits in the function of the amygdala have also been reported (Aleman and Kahn, 2005). Finally, schizophrenia is associated with broad cognitive impairment, including working and episodic memory, cognitive flexibility, and executive functions such as planning (Kahn and Keefe, 2013; McCutcheon et al., 2023). Defects in cognitive function have been primarily associated with disruption of cortical circuits in certain areas, most notably the dorsolateral prefrontal cortex, cingulate cortex, and hippocampal formation (Achim and Lepage, 2005; Fusar-Poli et al., 2007; Meyer-Lindenberg et al., 2005; Weinberger et al., 1986). Because working memory and other high-order cognitive functions require specific activity patterns across many of these regions (Goldman-Rakic, 1995), the mechanisms mediating the emergence of such patterns are likely key to understanding the neurobiological basis of schizophrenia.

## 2 Cortical PV+ interneurons

Information processing in the cerebral cortex critically depends on the precise spatial and temporal coordination of the activity of glutamatergic principal cells (also known as pyramidal cells) and GABAergic interneurons. Pyramidal cell activity is controlled by a rich diversity of interneurons, which control different temporal dynamics, the formation of neuronal ensembles, network oscillations, and brain states (Kepecs and Fishell, 2014; Klausberger and Somogyi, 2008).

Fast-spiking expressing the calcium-binding protein parvalbumin (PV+) cells are among the most abundant cortical interneurons. They have unique electrophysiological characteristics associated with high energy expenditure, including fast action potential kinetics and ion conductances (Table 1). In the context of this review, fast-spiking PV+ interneurons will primarily refer to PV+ basket cells. However, other interneurons, like chandelier cells and translaminar interneurons, also have fast-spiking characteristics and often express PV (Lim et al., 2018).

**Table 1:** Characteristics of cortical PV+ basket cells.

Property	Mechanism	Function
Parvalbumin expression	Binds Ca^2+^ with high-affinity	Calcium buffering
Perisomatic K^+^ channels	Kv3 potassium voltage-gated channels (KCNC1 and KCNC2)	Fast-spiking axon potentials
Fast excitatory postsynaptic currents	High density of excitatory synapses, rich in GRIA1 and GRIA4 AMPA receptor subunits	Coincidence detection
Fast inhibitory postsynaptic currents	Gamma-aminobutyric acid type A receptor subunit alpha1 (GABRA1)	High-frequency (gamma) network oscillations
High density of Na^2+^ channels in axons	Sodium voltage-gated alpha channels (SCN1A and SCN8A)	Fast axonal action potential propagation
Fast presynaptic Ca^2+^ channels	P/Q type Ca^2+^ channels (CACNA1A)	Fast and precise GABA release
Mutual inhibitory connectivity and autapses	Abundant PV-to-PV synapses	Adjust temporal firing intervals
Myelinated axons	Intermittent myelination	Mitochondria distribution? Trophic support?
High density of mitochondria in cell soma and axons	Energy supply	Support high energy demands, gamma oscillations
Protracted development	Activity-dependent maturation	Late onset of mature functional properties

PV+ interneurons can generate action potentials at high frequencies (>100 Hz) for prolonged periods with weak or no accommodation (Hu et al., 2014). Several key features support the fast-spiking behaviour of these cells. For example, their intrinsic membrane properties show resonance in the gamma-frequency band, and their ‘resting’ membrane potential is about 10–15 mV closer to the spike threshold than in pyramidal cells. In addition, their action potential has very high conduction velocity due to the high density of voltage-gated Na^+^ channels in the axon. The soma and dendrites of PV+ basket cells are densely covered with excitatory synapses (Gulyás et al., 1999), which facilitates the generation of fast excitatory postsynaptic potentials.

There are additional features that are characteristic of cortical PV+ interneurons. Most PV+ basket cells are enwrapped by a condensed form of extracellular matrix known as perineuronal nets (Fawcett et al., 2019). These structures regulate the contribution of PV+ interneurons to developmental critical periods and synaptic plasticity (Hensch, 2005; Reichelt et al., 2019). In addition, PV+ basket cells contain considerably higher numbers of mitochondria than other interneurons and pyramidal cells. Mitochondria in PV+ basket cells are also larger and enriched with proteins crucial for the electron transport chain, such as cytochrome c oxidase (complex IV) and cytochrome c (Fitzgerald et al., 2012; Gulyás et al., 2006; Takács et al., 2015).

The axons of PV+ basket cells are extensively arborised and make abundant perisomatic synapses on pyramidal cells (Freund and Buzsáki, 1996; Klausberger and Somogyi, 2008). This characteristic allows PV+ interneurons to tightly control the output of a local network by suppressing the generation of action potentials in principal cells. PV+ basket cells also exhibit mutual inhibitory innervation (Avermann et al., 2012; Bezaire and Soltesz, 2013; Galarreta and Hestrin, 1999; Gibson et al., 1999; Somogyi et al., 1998) and are frequently self-connected by autapses – synapses that a neuron makes onto itself – which allow them to adjust their temporal firing interval with extreme accuracy (Deleuze et al., 2019; Szegedi et al., 2020). Notably, the axons of PV+ basket cells are intermittently myelinated, a rather exceptional feature among cortical GABAergic interneurons in rodents and humans (Micheva et al., 2016; Stedehouder et al., 2017). Myelin function in PV+ cells remains unclear, but it has been suggested that myelin may provide trophic support to mitochondria in the axons of these cells to sustain high-frequency firing (Kole et al., 2022; Micheva et al., 2016).

The ability of PV+ interneurons to generate action potentials at high frequencies and their dense interconnectivity with pyramidal cells allows them to modulate network oscillations in the gamma frequency (∼30–80 Hz). Synchronous oscillations in neuronal activity in various frequency bands are essential for information processing and enable the coordinated function of different cortical areas. Gamma oscillations specifically increase during tasks requiring complex processing of sensory information, attention, and working and episodic memory (Fries, 2009; Griffiths and Jensen, 2023; Ni et al., 2016; Uhlhaas and Singer, 2012), and fast-spiking interneurons are uniquely required for their generation and maintenance (Buzsáki and Wang, 2012; Cardin et al., 2009; Sohal et al., 2009).

## 3 Developmental and metabolic vulnerability of PV+ interneurons

PV+ interneurons follow a very protracted period of maturation that enables the fast-spiking properties of these cells (Doischer et al., 2008; Goldberg et al., 2011). This process occurs concurrently with an increase in the density of glutamate synapses received by these cells (Miyamae et al., 2017), suggesting that excitatory inputs shape the maturation of PV+ basket cells. Indeed, PV levels correlate with the degree of maturation of glutamatergic synapses contacting PV+ interneurons (Chung et al., 2017). Perineuronal nets are also strongly regulated during development, increasing with the neurochemical and physiological maturation of PV+ fast-spiking interneurons (Condé et al., 1996; Nowicka et al., 2009; Reynolds and Beasley, 2001; Rio et al., 1994; Rogers et al., 2018; Ye and Miao, 2013). Recent experiments suggest that early network dynamics in the mouse postnatal cortex prevent the premature maturation of PV+ interneurons (Mòdol et al., 2023). In the adult cortex, the density of glutamatergic synapses targeting PV+ interneurons predicts cellular PV levels in both mice and humans (Chung et al., 2016; Donato et al., 2013). Because PV+ basket cells might not fully mature in rodents and primates until puberty (Fung et al., 2010; Miyamae et al., 2017), their contribution to cognitive function during adolescence and early adult life might be significantly different than in adulthood.

The protracted development of PV+ basket cells and the sensitivity of this process to neuronal activity make these cells particularly susceptible to schizophrenia risk factors, such as early life adversity and social isolation. For example, early life stress affects the development of PV+ interneurons in the mouse prefrontal cortex (Goodwill et al., 2018). The function of PV+ interneurons in the adult cortex is also particularly susceptible to changes in neuronal activity during adolescence (Canetta et al., 2022). Consistently, social interactions during this period strongly influence the function of cortical PV+ interneurons in mice (Jeon et al., 2023).

PV+ interneurons have considerable metabolic demands due to their electrophysiological characteristics. Energy is required for the dissipation of the strong ion gradients caused by the abundant excitatory and inhibitory inputs PV+ basket cells receive, their ability to sustain high-frequency firing and contribute to gamma oscillations, and the sizeable quantal size and high probability of GABA release of these cells (Kann, 2016). PV+ interneurons can meet these high energy demands due to their elevated mitochondrial content, but this also renders these cells more susceptible to oxidative stress and alterations of mitochondrial function.

## 4 Cortical PV interneuron deficits in schizophrenia

Although GABAergic deficits in schizophrenia may implicate several types of interneurons, the reduction in specific markers suggests a prominent involvement of fast-spiking basket cells in the pathophysiology of the disorder (Table 2). For instance, the levels of GAD67, PV, and individual components of perineuronal nets are specifically reduced in PV+ basket cells in schizophrenia patients across multiple cortical areas (Fung et al., 2010; Glausier et al., 2014; Hashimoto et al., 2008, 2003; Mauney et al., 2013; Zhang and Reynolds, 2002). Importantly, this reduction is not due to a decrease in the density of PV+ interneurons but rather in the levels of these critical proteins (Enwright et al., 2016; Hashimoto et al., 2003). Because the expression of PV and GAD67 is activity-dependent, it has been suggested that a reduction in the excitatory drive of PV+ basket cells may account for these observations. In agreement with this idea, the density of excitatory synapses contacting PV+ interneurons in the prefrontal cortex is lower in schizophrenia patients than in controls (Chung et al., 2016).

**Table 2:** Alterations of cortical PV+ interneurons in schizophrenia.

Alteration	Type of study	Reference
Reduced PV levels	Human postmortem	Enwright et al., 2016; Fung et al., 2010; Glausier et al., 2014; Hashimoto et al., 2003; Hashimoto et al., 2008; Zhang and Reynolds, 2002
	Mouse models	Belforte et al., 2010; Pino et al., 2013
Reduced GAD67 levels	Human postmortem	Hashimoto et al., 2003
	Mouse models	Belforte et al., 2010; Pino et al., 2013
Reduce perineuronal nets	Human postmortem	Enwright et al., 2016; Mauney et al., 2013
	Mouse models	Steullet et al., 2017
Reduced excitatory synapses	Human postmortem	Chung et al., 2016
	Mouse models	Fazzari et al., 2010; Pino et al., 2013; Ting et al., 2011
Abnormal gamma oscillations	EEG and MEG in humans	Cho et al., 2006; Gallinat et al., 2004; Kikuchi et al., 2011; Kwon et al., 1999; Light et al., 2006; Reilly et al., 2018; Thuné et al., 2016; Tikka et al., 2013; Yadav et al., 2021
	Mouse models	Carlén et al., 2011; Korotkova et al., 2010; Pino et al., 2013

The reduction in the levels of PV and GAD67 in schizophrenia has been interpreted as a reduction in the activity of PV+ interneurons because the expression of these markers is strongly linked to the activity of PV+ basket cells. Since the inhibition of pyramidal cells by PV+ basket cells is required for the generation of gamma oscillations (Metzner et al., 2016; Vierling-Claassen et al., 2008), a reduction in the activity of these interneurons should lead to deficits in neural synchrony in the cerebral cortex. Consistently, abnormalities in gamma frequency oscillations have been described across multiple cortical areas in schizophrenic patients (Cho et al., 2006; Gallinat et al., 2004; Gonzalez-Burgos et al., 2015; Kikuchi et al., 2011; Kwon et al., 1999; Light et al., 2006; Uhlhaas and Singer, 2010). For example, the power of gamma oscillations in the prefrontal cortex increases during working memory tasks in healthy volunteers but not in schizophrenia patients (Cho et al., 2006). Oscillatory activity can also be probed in humans independently of a task, for instance, by recording steady-state responses to trains of sensory stimuli at different frequencies. Schizophrenia patients consistently exhibit reduced power and phase delay to 40-Hz auditory stimulation (Kwon et al., 1999; Thuné et al., 2016), suggesting that deficits in neural synchrony in the gamma range are pervasive in schizophrenia. Significantly, drug-naïve patients with first-episode psychosis may have increased resting state gamma power across multiple cortical regions (Reilly et al., 2018; Tikka et al., 2013; Yadav et al., 2021).

Mitochondrial function has long been suggested to influence the development and course of schizophrenia (Clay et al., 2011; Cuenod et al., 2022; Hjelm et al., 2015). Mitochondria play critical roles in cellular bioenergetics, calcium homeostasis, redox signalling, and apoptosis (Friedman and Nunnari, 2014). Postmortem studies have identified a reduction in the expression of genes encoding mitochondrial complexes in schizophrenia (Arion et al., 2015; Glausier et al., 2020; Karry et al., 2004; Prabakaran et al., 2004), and similar abnormalities have been detected in iPSC-derived cortical neurons from schizophrenia patients (Brennand et al., 2015; Kathuria et al., 2020; Ni et al., 2020). Moreover, excitatory neurons derived from individuals with 22q11.2 deletion syndrome and schizophrenia, but not those without schizophrenia, have mitochondrial dysfunction (Li et al., 2021, 2019). Neurons derived from 22q11.2 deletion syndrome individuals without schizophrenia have increased mitochondrial function compared to control neurons (Li et al., 2021), suggesting that the development of schizophrenia in 22q11.2 deletion syndrome patients – and perhaps other individuals at high clinical risk for psychosis – might be influenced by compensatory mitochondrial function. This observation is consistent with genome-wide association studies suggesting that genetic variants related to bioenergetics may increase the risk of schizophrenia (Ripke et al., 2014; Trubetskoy et al., 2022). However, alterations in gene expression related to mitochondrial function seem more prominent in pyramidal cells than in PV+ basket cells (Enwright et al., 2017).

## 5 Preclinical studies linking PV interneuron dysfunction and schizophrenia

Experiments in mice have demonstrated that disrupting the development of PV+ interneurons can reproduce many pathophysiological observations made in schizophrenia patients. For instance, work from several laboratories has revealed that signalling downstream of the Erb-B2 receptor tyrosine kinase 4 (ErbB4) controls the formation of excitatory synapses on PV+ interneurons (Exposito-Alonso et al., 2020; Fazzari et al., 2010; Müller et al., 2018; Pino et al., 2013; Ting et al., 2011). ErbB4 activation leads to the expression and local translation of a molecular programme required to form glutamatergic synapses onto PV+ interneurons (Bernard et al., 2022). Consistently, conditional deletion of ErbB4 from PV+ interneurons reduces the density of glutamatergic synapses contacting PV+ basket cells (Pino et al., 2013). This alteration causes a prominent decrease in the expression of PV and GAD67, impairs the recruitment of PV+ interneurons, disrupts gamma oscillations in the hippocampus and prefrontal cortex, and perturbs cognitive function in mice (Pino et al., 2013). Loss of ErbB4 in PV+ interneurons also causes a non-cell autonomous reduction in the density of dendritic spines in pyramidal cells (Pino et al., 2013; Yin et al., 2013), suggesting that this characteristic pathological feature of schizophrenia might be – at least theoretically – a secondary adaptation to defective inhibition in the cerebral cortex. Importantly, ErbB4 expression in PV+ interneurons is conserved in rodents and primates, including humans (Neddens et al., 2011). Rare mutations and tandem repeat expansion in *ERBB4* have been linked to schizophrenia and intellectual disability (Kasnauskiene et al., 2013; Mojarad et al., 2022; Walsh et al., 2008), and common genetic variation in this locus also seems to be associated with the disorder (Marenco et al., 2011; Pardiñas et al., 2018; Trubetskoy et al., 2022). Dysregulated alternative splicing of *ERBB4* has also been reported in the cerebral cortex of schizophrenia patients (Chung et al., 2015). Irrespectively, these studies suggest that the defective development of excitatory synapses contacting PV+ interneurons is a plausible pathophysiological mechanism in schizophrenia.

Mouse genetic studies investigating the function of N-methyl-D-aspartate (NMDA) receptors in PV+ interneurons further support the idea that disrupting the excitatory drive these cells receive from pyramidal cells impacts their function in mature circuits. For example, deletion of the mandatory GRIN1 subunit of NMDA receptors from interneurons during early postnatal development results in reduced expression in PV and GAD67, disinhibition of pyramidal cells, and abnormal neural synchrony in the mouse cerebral cortex (Belforte et al., 2010). Although these genetic experiments may involve interneurons other than PV+ basket cells, conditional mutants deleting GRIN1 exclusively from PV+ interneurons also exhibit enhanced baseline gamma rhythms and impaired cognitive function (Carlén et al., 2011; Korotkova et al., 2010). Genetic variation in *GRIN1* has not been associated with schizophrenia, but rare mutations in *GRIN2A*, which encodes another subunit of NMDA receptors, increase the risk of developing schizophrenia by almost 20-fold (Singh et al., 2022).

One important consideration regarding the findings linking defects in the glutamatergic recruitment of PV+ interneurons and the contribution of these cells to the pathophysiology of schizophrenia is timing. Early deletion of ErbB4 and GRIN1 from PV+ interneurons produces molecular and behavioural abnormalities that are not observed if the loss of these proteins occurs after adolescence (Batista-Brito et al., 2023; Belforte et al., 2010; Mallien et al., 2021). Consistently, adult re-expression of ErbB4 only partially reverses the phenotypes observed following the developmental deletion of this protein (Wang et al., 2018). Moreover, the contribution of NMDA currents in immature PV+ interneurons is very significant but subsequently declines during postnatal development until reaching low levels in adulthood (Wang and Gao, 2009, 2010). These observations emphasise the unique role of ErbB4 and NMDA receptors in the maturation and wiring of PV+ interneurons and suggest that developmental disruption of cortical inhibitory circuits mediated by PV+ interneurons is central to the pathophysiology of schizophrenia.

Beyond alterations in the afferent connectivity of PV+ interneurons, other mechanisms may account for the dysfunction of these cells in schizophrenia. For instance, genetic and pharmacological manipulations of the redox system significantly impact PV+ interneurons (Cuenod et al., 2022). This observation is consistent with the notion that PV+ interneurons have high energy demands satisfied by oxidative phosphorylation at mitochondria, making these cells particularly vulnerable to redox imbalance. Interestingly, PV+ interneurons are more susceptible to redox dysregulation during early postnatal development rather than later in life (Cabungcal et al., 2013a, 2014). This might be due to the immaturity of perineuronal nets, which seem to protect PV+ interneurons from oxidative stress (Cabungcal et al., 2013b). Oxidative stress has been reported in many animal models of neurodevelopmental disorders, leading to the suggestion that redox dysregulation might be upstream of PV+ interneuron deficits in schizophrenia (Steullet et al., 2017). This hypothesis requires additional support from clinical studies.

PV+ interneurons may also be particularly susceptible to myelin pathology due to the small diameter of their axons. In agreement with this notion, experimental demyelination disrupts inhibitory transmission by PV+ interneurons, compromising gamma oscillations (Dubey et al., 2022). Myelin also seems to regulate the spatial distribution of mitochondria in the axons of PV+ basket cells, which tend to accumulate in the myelinated segments (Kole et al., 2022). Intriguingly, disrupting mitochondria trafficking in PV+ interneurons reduces their density in axons and severely impairs gamma oscillations (Kontou et al., 2021). Similarly, experimental demyelination of PV+ interneurons disrupts gamma oscillations in vivo (Dubey et al., 2022). Although myelination deficits have been extensively implicated in schizophrenia (Fitzsimmons et al., 2013), and effect sizes are more significant in the grey matter than in the white matter (Miyakawa et al., 1972; Uranova et al., 2011), specific defects in PV+ interneurons in the cortex of schizophrenia patients have not yet been reported.

## 6 Conclusions

The dysfunction of PV+ interneurons is a consistent finding in schizophrenia, but the underlying pathophysiological mechanisms remain ambiguous. Considering the close relationship between pyramidal cells and PV+ interneurons in cortical circuits, it is conceivable that multiple primary alterations can lead to the same changes in GABAergic markers observed in schizophrenia patients. As summarised above, many of the alterations observed in the cortex of schizophrenia patients, including non-cell autonomous defects in pyramidal cells, can be caused by primary alterations in PV+ interneurons. However, it is equally conceivable that all these changes arise secondary to a primary problem in pyramidal cells. For example, genetic variation may primarily reduce glutamatergic transmission among pyramidal cells, and this could, in turn, lead to a secondary reduction in the activity of PV+ basket cells (Dienel et al., 2022). Therefore, there might not be a unique, common pathway leading to the characteristic alterations of the cerebral cortex in schizophrenia patients. Nonetheless, the dysfunction of this critical inhibitory motif seems unequivocally linked with the disruption of neuronal synchrony in the gamma range and the accompanying defects in cognitive function, independently of the causal mechanism.

It is worth emphasising that the mechanisms mediating the onset of psychosis are likely different across patient populations. NMDA receptor antagonists and autoimmune encephalitis caused by antibodies against NMDA receptor subunits can trigger psychosis in adults without a history of developmental problems (Fukata et al., 2018; Moghaddam and Krystal, 2012). In contrast, experiments in mice indicate that alterations in the developmental trajectory of PV+ interneurons likely drive the deficits observed in the adult cortex. It is still unclear why such early developmental problems give rise to cognitive and social problems during childhood and early adolescence and eventually translate into a psychotic disorder in early adulthood. One possibility is that alterations in cortical circuits cause pathological maladaptation in other brain circuits, including the dopaminergic system, which would explain why cognitive deficits predate the onset of psychosis in schizophrenia. Although some experimental evidence supports this idea (Howes and Shatalina, 2022), future experiments should aim to identify a causal link between the alteration of cortical PV+ interneurons and striatal hyperdopaminergia.

## Contributions

O.M. wrote this review.

## Conflict of Interest Statement

The author has no conflict of interest to declare.

## References

[bib0001] Achim A.M., Lepage M. (2005). Episodic memory-related activation in schizophrenia: meta-analysis. Br. J. Psychiatry.

[bib0002] Aleman A., Kahn R.S. (2005). Strange feelings: Do amygdala abnormalities dysregulate the emotional brain in schizophrenia?. Prog. Neurobiol..

[bib0003] Arion D., Corradi J.P., Tang S., Datta D., Boothe F., He A., Cacace A.M., Zaczek R., Albright C.F., Tseng G., Lewis D.A. (2015). Distinctive transcriptome alterations of prefrontal pyramidal neurons in schizophrenia and schizoaffective disorder. Mol. Psychiatry.

[bib0004] Avermann M., Tomm C., Mateo C., Gerstner W., Petersen C.C.H. (2012). Microcircuits of excitatory and inhibitory neurons in layer 2/3 of mouse barrel cortex. J. Neurophysiol..

[bib0005] Batista-Brito R., Majumdar A., Nuño A., Ward C., Barnes C., Nikouei K., Vinck M., Cardin J.A. (2023). Developmental loss of ErbB4 in PV interneurons disrupts state-dependent cortical circuit dynamics. Mol. Psychiatry.

[bib0006] Belforte J.E., Zsiros V., Sklar E.R., Jiang Z., Yu G., Li Y., Quinlan E.M., Nakazawa K. (2010). Postnatal NMDA receptor ablation in corticolimbic interneurons confers schizophrenia-like phenotypes. Nat. Neurosci..

[bib0007] Bernard C., Exposito-Alonso D., Selten M., Sanalidou S., Hanusz-Godoy A., Aguilera A., Hamid F., Oozeer F., Maeso P., Allison L., Russell M., Fleck R.A., Rico B., Marín O. (2022). Cortical wiring by synapse type–specific control of local protein synthesis. Science.

[bib0008] Bezaire M.J., Soltesz I. (2013). Quantitative assessment of CA1 local circuits: Knowledge base for interneuron-pyramidal cell connectivity. Hippocampus.

[bib0009] Brennand K., Savas J.N., Kim Y., Tran N., Simone A., Hashimoto-Torii K., Beaumont K.G., Kim H.J., Topol A., Ladran I., Abdelrahim M., Matikainen-Ankney B., Chao S., Mrksich M., Rakic P., Fang G., Zhang B., Yates J.R., Gage F.H. (2015). Phenotypic differences in hiPSC NPCs derived from patients with schizophrenia. Mol. Psychiatry.

[bib0010] Buzsáki G., Wang X.-J. (2012). Mechanisms of Gamma Oscillations. Annu. Rev. Neurosci..

[bib0011] Cabungcal J.-H., Counotte D.S., Lewis E.M., Tejeda H.A., Piantadosi P., Pollock C., Calhoon G.G., Sullivan E.M., Presgraves E., Kil J., Hong L.E., Cuenod M., Do K.Q., O'Donnell P. (2014). Juvenile Antioxidant Treatment Prevents Adult Deficits in a Developmental Model of Schizophrenia. Neuron.

[bib0012] Cabungcal J.-H., Steullet P., Kraftsik R., Cuenod M., Do K.Q. (2013). Early-life insults impair parvalbumin interneurons via oxidative stress: reversal by N-acetylcysteine. Biol. Psychiatry.

[bib0013] Cabungcal J.-H., Steullet P., Morishita H., Kraftsik R., Cuenod M., Hensch T.K., Do K.Q. (2013). Perineuronal nets protect fast-spiking interneurons against oxidative stress. Proc. Natl. Acad. Sci. USA.

[bib0014] Canetta S.E., Holt E.S., Benoit L.J., Teboul E., Sahyoun G.M., Ogden R.T., Harris A.Z., Kellendonk C. (2022). Mature parvalbumin interneuron function in prefrontal cortex requires activity during a postnatal sensitive period. Elife.

[bib0015] Cannon M., Jones P.B., Murray R.M. (2002). Obstetric Complications and Schizophrenia: Historical and Meta-Analytic Review. Am. J. Psychiatry.

[bib0016] Cardin J.A., Carlén M., Meletis K., Knoblich U., Zhang F., Deisseroth K., Tsai L.-H., Moore C.I. (2009). Driving fast-spiking cells induces gamma rhythm and controls sensory responses. Nature.

[bib0017] Carlén M., Meletis K., Siegle J.H., Cardin J.A., Futai K., Vierling-Claassen D., Rühlmann C., Jones S.R., Deisseroth K., Sheng M., Moore C.I., Tsai L.-H. (2011). A critical role for NMDA receptors in parvalbumin interneurons for gamma rhythm induction and behavior. Mol. Psychiatry..

[bib0018] Carpenter W.T., Buchanan R.W. (1994). Schizophrenia. N. Engl. J. Med..

[bib0019] Cho R.Y., Konecky R.O., Carter C.S. (2006). Proc. Natl. Acad. Sci. USA.

[bib0020] Chung D.W., Fish K.N., Lewis D.A. (2016). Pathological Basis for Deficient Excitatory Drive to Cortical Parvalbumin Interneurons in Schizophrenia. Am. J. Psychiatry.

[bib0021] Chung D.W., Volk D.W., Arion D., Zhang Y., Sampson A.R., Lewis D.A. (2015). Dysregulated ErbB4 Splicing in Schizophrenia: Selective Effects on Parvalbumin Expression. Am. J. Psychiatry.

[bib0022] Chung D.W., Wills Z.P., Fish K.N., Lewis D.A. (2017). Developmental pruning of excitatory synaptic inputs to parvalbumin interneurons in monkey prefrontal cortex. Proc. Natl. Acad. Sci. USA.

[bib0023] Clay H.B., Sillivan S., Konradi C. (2011). Mitochondrial dysfunction and pathology in bipolar disorder and schizophrenia. Int. J. Dev. Neurosci..

[bib0024] Condé F., Lund J.S., Lewis D.A. (1996). The hierarchical development of monkey visual cortical regions as revealed by the maturation of parvalbumin-immunoreactive neurons. Dev. Brain Res..

[bib0025] Cuenod M., Steullet P., Cabungcal J.-H., Dwir D., Khadimallah I., Klauser P., Conus P., Do K.Q. (2022). Caught in vicious circles: a perspective on dynamic feed-forward loops driving oxidative stress in schizophrenia. Mol. Psychiatry.

[bib0026] Deleuze C., Bhumbra G.S., Pazienti A., Lourenço J., Mailhes C., Aguirre A., Beato M., Bacci A. (2019). Strong preference for autaptic self-connectivity of neocortical PV interneurons facilitates their tuning to γ-oscillations. PLoS Biol.

[bib0027] Dienel S.J., Lewis D.A. (2019). Alterations in cortical interneurons and cognitive function in schizophrenia. Neurobiol. Dis..

[bib0028] Dienel S.J., Schoonover K.E., Lewis D.A. (2022). Cognitive Dysfunction and Prefrontal Cortical Circuit Alterations in Schizophrenia: Developmental Trajectories. Biol. Psychiatry.

[bib0029] Doischer D., Hosp J.A., Yanagawa Y., Obata K., Jonas P., Vida I., Bartos M. (2008). Postnatal Differentiation of Basket Cells from Slow to Fast Signaling Devices. J. Neurosci..

[bib0030] Donato F., Rompani S.B., Caroni P. (2013). Parvalbumin-expressing basket-cell network plasticity induced by experience regulates adult learning. Nature.

[bib0031] Dubey, M., Pascual-Garcia, M., Helmes, K., Wever, D.D., Hamada, M.S., Kushner, S.A., Kole, M.H., 2022. Myelination synchronizes cortical oscillations by consolidating parvalbumin-mediated phasic inhibition. eLife 11, e73827. 10.7554/elife.73827. PMC8887893 35001871

[bib0032] Enwright J.F., Sanapala S., Foglio A., Berry R., Fish K.N., Lewis D.A. (2016). Reduced Labeling of Parvalbumin Neurons and Perineuronal Nets in the Dorsolateral Prefrontal Cortex of Subjects with Schizophrenia. Neuropsychopharmacology.

[bib0033] Enwright J.F., Huo Z., Arion D., Corradi J.P., Tseng G., Lewis D.A. (2017). Transcriptome alterations of prefrontal cortical parvalbumin neurons in schizophrenia. Mol. Psychiatry.

[bib0034] Exposito-Alonso D., Osório C., Bernard C., Pascual-García S., Pino I.del, Marin O., Rico B. (2020). Subcellular sorting of neuregulins controls the assembly of excitatory-inhibitory cortical circuits. eLife.

[bib0035] Fawcett J.W., Oohashi T., Pizzorusso T. (2019). The roles of perineuronal nets and the perinodal extracellular matrix in neuronal function. Nat. Rev. Neurosci..

[bib0036] Fazzari P., Paternain A.V., Valiente M., Pla R., Luján R., Lloyd K., Lerma J., Marin O., Rico B. (2010). Control of cortical GABA circuitry development by Nrg1 and ErbB4 signalling. Nature.

[bib0037] Fitzgerald M.L., Chan J., Mackie K., Lupica C.R., Pickel V.M. (2012). Altered dendritic distribution of dopamine D2 receptors and reduction in mitochondrial number in parvalbumin-containing interneurons in the medial prefrontal cortex of cannabinoid-1 (CB1) receptor knockout mice. J. Comp. Neurol..

[bib0038] Fitzsimmons J., Kubicki M., Shenton M.E. (2013). Review of functional and anatomical brain connectivity findings in schizophrenia. Curr. Opin. Psychiatry.

[bib0039] Freund T.F., Buzsáki G. (1996). Interneurons of the hippocampus. Hippocampus.

[bib0040] Friedman J.R., Nunnari J. (2014). Mitochondrial form and function. Nature.

[bib0041] Fries P. (2009). Neuronal Gamma-Band Synchronization as a Fundamental Process in Cortical Computation. Annu. Rev. Neurosci..

[bib0042] Fukata M., Yokoi N., Fukata Y. (2018). Neurobiology of autoimmune encephalitis. Curr. Opin. Neurobiol..

[bib0043] Fung S.J., Webster M.J., Sivagnanasundaram S., Duncan C., Elashoff M., Weickert C.S. (2010). Expression of Interneuron Markers in the Dorsolateral Prefrontal Cortex of the Developing Human and in Schizophrenia. Am. J. Psychiatry.

[bib0044] Fusar-Poli P., Perez J., Broome M., Borgwardt S., Placentino A., Caverzasi E., Cortesi M., Veggiotti P., Politi P., Barale F., McGuire P. (2007). Neurofunctional correlates of vulnerability to psychosis: A systematic review and meta-analysis. Neurosci. Biobehav. Rev..

[bib0045] Galarreta M., Hestrin S. (1999). A network of fast-spiking cells in the neocortex connected by electrical synapses. Nature.

[bib0046] Gallinat J., Winterer G., Herrmann C.S., Senkowski D. (2004). Reduced oscillatory gamma-band responses in unmedicated schizophrenic patients indicate impaired frontal network processing. Clin. Neurophysiol..

[bib0047] Gibson J.R., Beierlein M., Connors B.W. (1999). Two networks of electrically coupled inhibitory neurons in neocortex. Nature.

[bib0048] Glausier J.R., Enwright J.F., Lewis D.A. (2020). Diagnosis- and Cell Type-Specific Mitochondrial Functional Pathway Signatures in Schizophrenia and Bipolar Disorder. Am. J. Psychiatry.

[bib0049] Glausier J.R., Fish K.N., Lewis D.A. (2014). Altered parvalbumin basket cell inputs in the dorsolateral prefrontal cortex of schizophrenia subjects. Mol. Psychiatry.

[bib0050] Goldberg E.M., Jeong H.-Y., Kruglikov I., Tremblay R., Lazarenko R.M., Rudy B. (2011). Rapid developmental maturation of neocortical FS cell intrinsic excitability. Cereb. Cortex.

[bib0051] Goldman-Rakic P.S. (1995). Cellular basis of working memory. Neuron.

[bib0052] Gonzalez-Burgos G., Cho R.Y., Lewis D.A. (2015). Alterations in Cortical Network Oscillations and Parvalbumin Neurons in Schizophrenia. Biol. Psychiatry.

[bib0053] Goodwill H.L., Manzano-Nieves G., LaChance P., Teramoto S., Lin S., Lopez C., Stevenson R.J., Theyel B.B., Moore C.I., Connors B.W., Bath K.G. (2018). Early Life Stress Drives Sex-Selective Impairment in Reversal Learning by Affecting Parvalbumin Interneurons in Orbitofrontal Cortex of Mice. Cell Rep.

[bib0054] Griffiths B.J., Jensen O. (2023). Gamma oscillations and episodic memory. Trends Neurosci.

[bib0055] Gulyás A.I., Buzsáki G., Freund T.F., Hirase H. (2006). Populations of hippocampal inhibitory neurons express different levels of cytochrome c. Eur. J. Neurosci..

[bib0056] Gulyás A.I., Megías M., Emri Z., Freund T.F. (1999). Total number and ratio of excitatory and inhibitory synapses converging onto single interneurons of different types in the CA1 area of the rat hippocampus. J. Neurosci..

[bib0057] Hashimoto T., Unger T., Maldonado-Avilés J.G., Morris H.M., Volk D.W., Mirnics K., Lewis D.A. (2008). Alterations in GABA-related transcriptome in the dorsolateral prefrontal cortex of subjects with schizophrenia. Mol. Psychiatry.

[bib0058] Hashimoto T., Volk D.W., Eggan S.M., Mirnics K., Pierri J.N., Sun Z., Sampson A.R., Lewis D.A. (2003). Gene Expression Deficits in a Subclass of GABA Neurons in the Prefrontal Cortex of Subjects with Schizophrenia. J. Neurosci..

[bib0059] Hensch T.K. (2005). Critical period plasticity in local cortical circuits. Nat. Rev. Neurosci..

[bib0060] Hjelm B.E., Rollins B., Mamdani F., Lauterborn J.C., Kirov G., Lynch G., Gall C.M., Sequeira A., Vawter M.P. (2015). Evidence of Mitochondrial Dysfunction within the Complex Genetic Etiology of Schizophrenia. Mol. Neuropsychiatry.

[bib0061] Howes O.D., Kambeitz J., Kim E., Stahl D., Slifstein M., Abi-Dargham A., Kapur S. (2012). The Nature of Dopamine Dysfunction in Schizophrenia and What This Means for Treatment. Arch. Gen. Psychiatry.

[bib0062] Howes O.D., Murray R.M. (2014). Schizophrenia: an integrated sociodevelopmental-cognitive model. Lancet.

[bib0063] Howes O.D., Shatalina E. (2022). Integrating the Neurodevelopmental and Dopamine Hypotheses of Schizophrenia and the Role of Cortical Excitation-Inhibition Balance. Biol. Psychiatry.

[bib0064] Hu H., Gan J., Jonas P. (2014). Fast-spiking, parvalbumin^+^ GABAergic interneurons: from cellular design to microcircuit function. Science.

[bib0065] Hu W., MacDonald M.L., Elswick D.E., Sweet R.A. (2015). The glutamate hypothesis of schizophrenia: evidence from human brain tissue studies. Ann. N. York Acad. Sci..

[bib0066] Hyde T.M., Deep-Soboslay A., Iglesias B., Callicott J.H., Gold J.M., Meyer-Lindenberg A., Honea R.A., Bigelow L.B., Egan M.F., Emsellem E.M., Weinberger D.R. (2008). Enuresis as a premorbid developmental marker of schizophrenia. Brain.

[bib0067] Jääskeläinen E., Miettunen J., Veijola J., McGrath J.J., Murray G.K., Jones P.B., Isohanni M. (2008). Associations between early development and outcome in schizophrenia — A 35-year follow-up of the Northern Finland 1966 Birth Cohort. Schizophr. Res..

[bib0068] Jeon Y.-S., Jeong D., Kweon H., Kim J.-H., Kim C.Y., Oh Y., Lee Y.-H., Kim C.H., Kim S.-G., Jeong J.-W., Kim E., Lee S.-H. (2023). Adolescent Parvalbumin Expression in the Left Orbitofrontal Cortex Shapes Sociability in Female Mice. J. Neurosci..

[bib0069] Juckel G., Schlagenhauf F., Koslowski M., Wüstenberg T., Villringer A., Knutson B., Wrase J., Heinz A. (2006). Dysfunction of ventral striatal reward prediction in schizophrenia. NeuroImage.

[bib0070] Kahn R.S., Keefe R.S.E. (2013). Schizophrenia Is a Cognitive Illness: Time for a Change in Focus. JAMA Psychiatry.

[bib0071] Kahn R.S., Sommer I.E., Murray R.M., Meyer-Lindenberg A., Weinberger D.R., Cannon T.D., O'Donovan M., Correll C.U., Kane J.M., Os J.van, Insel T.R. (2015). Schizophrenia. Nat. Rev. Dis. Prim..

[bib0072] Kann O. (2016). The interneuron energy hypothesis: Implications for brain disease. Neurobiol. Dis..

[bib0073] Karry R., Klein E., Shachar D.B. (2004). Mitochondrial complex i subunits expression is altered in schizophrenia: a postmortem study. Biol. Psychiatry.

[bib0074] Kasnauskiene J., Ciuladaite Z., Preiksaitiene E., Utkus A., Peciulyte A., Kučinskas V. (2013). A New Single Gene Deletion on 2q34: ERBB4 Is Associated With Intellectual Disability. Am. J. Med. Genet. A.

[bib0075] Kathuria A., Lopez-Lengowski K., Jagtap S.S., McPhie D., Perlis R.H., Cohen B.M., Karmacharya R. (2020). Transcriptomic Landscape and Functional Characterization of Induced Pluripotent Stem Cell–Derived Cerebral Organoids in Schizophrenia. JAMA Psychiatry.

[bib0076] Kepecs A., Fishell G. (2014). Interneuron cell types are fit to function. Nature.

[bib0077] Kesby J., Eyles D., McGrath J., Scott J. (2018). Dopamine, psychosis and schizophrenia: the widening gap between basic and clinical neuroscience. Transl. Psychiatry.

[bib0078] Kikuchi M., Hashimoto T., Nagasawa T., Hirosawa T., Minabe Y., Yoshimura M., Strik W., Dierks T., Koenig T. (2011). Frontal areas contribute to reduced global coordination of resting-state gamma activities in drug-naïve patients with schizophrenia. Schizophr. Res..

[bib0079] Klausberger T., Somogyi P. (2008). Neuronal diversity and temporal dynamics: the unity of hippocampal circuit operations. Science.

[bib0080] Kole K., Voesenek B.J.B., Brinia M.E., Petersen N., Kole M.H.P. (2022). Parvalbumin basket cell myelination accumulates axonal mitochondria to internodes. Nat. Commun..

[bib0081] Kontou G., Antonoudiou P., Podpolny M., Szulc B.R., Arancibia-Carcamo I.L., Higgs N.F., Lopez-Domenech G., Salinas P.C., Mann E.O., Kittler J.T. (2021). Miro1-dependent mitochondrial dynamics in parvalbumin interneurons. eLife.

[bib0082] Korotkova T., Fuchs E.C., Ponomarenko A., Engelhardt J.von, Monyer H. (2010). NMDA receptor ablation on parvalbumin-positive interneurons impairs hippocampal synchrony, spatial representations, and working memory. Neuron.

[bib0083] Kwon J.S., O'Donnell B.F., Wallenstein G.V., Greene R.W., Hirayasu Y., Nestor P.G., Hasselmo M.E., Potts G.F., Shenton M.E., McCarley R.W. (1999). Gamma Frequency–Range Abnormalities to Auditory Stimulation in Schizophrenia. Arch. Gen. Psychiatry.

[bib0084] Li J., Ryan S.K., Deboer E., Cook K., Fitzgerald S., Lachman H.M., Wallace D.C., Goldberg E.M., Anderson S.A. (2019). Mitochondrial deficits in human iPSC-derived neurons from patients with 22q11.2 deletion syndrome and schizophrenia. Transl. Psychiatry.

[bib0085] Li J., Tran O.T., Crowley T.B., Moore T.M., Zackai E.H., Emanuel B.S., McDonald-McGinn D.M., Gur R.E., Wallace D.C., Anderson S.A. (2021). Association of Mitochondrial Biogenesis With Variable Penetrance of Schizophrenia. JAMA Psychiatry.

[bib0086] Light G.A., Hsu J.L., Hsieh M.H., Meyer-Gomes K., Sprock J., Swerdlow N.R., Braff D.L. (2006). Gamma Band Oscillations Reveal Neural Network Cortical Coherence Dysfunction in Schizophrenia Patients. Biol. Psychiatry.

[bib0087] Lim L., Mi D., Llorca A., Marín O. (2018). Development and Functional Diversification of Cortical Interneurons. Neuron.

[bib0088] Lyon G.J., Abi-Dargham A., Moore H., Lieberman J.A., Javitch J.A., Sulzer D. (2011). Presynaptic Regulation of Dopamine Transmission in Schizophrenia. Schizophr. Bull..

[bib0089] Mallien A.S., Pfeiffer N., Vogt M.A., Chourbaji S., Sprengel R., Gass P., Inta D. (2021). Cre-Activation in ErbB4-Positive Neurons of Floxed Grin1/NMDA Receptor Mice Is Not Associated With Major Behavioral Impairment. Front. Psychiatry.

[bib0090] Marenco S., Geramita M., Veen J.W.van der, Barnett A.S., Kolachana B., Shen J., Weinberger D.R., Law A.J. (2011). Genetic association of ErbB4 and human cortical GABA levels in vivo. J. Neurosci..

[bib0091] Mauney S.A., Athanas K.M., Pantazopoulos H., Shaskan N., Passeri E., Berretta S., Woo T.-U.W. (2013). Developmental Pattern of Perineuronal Nets in the Human Prefrontal Cortex and Their Deficit in Schizophrenia. Biol. Psychiatry.

[bib0092] McCutcheon R.A., Abi-Dargham A., Howes O.D. (2019). Schizophrenia, Dopamine and the Striatum: From Biology to Symptoms. Trends Neurosci..

[bib0093] McCutcheon R.A., Keefe R.S.E., McGuire P.K. (2023). Cognitive impairment in schizophrenia: aetiology, pathophysiology, and treatment. Mol. Psychiatry.

[bib0094] Metzner C., Schweikard A., Zurowski B. (2016). Multifactorial Modeling of Impairment of Evoked Gamma Range Oscillations in Schizophrenia. Front. Comput. Neurosci..

[bib0095] Meyer-Lindenberg A.S., Olsen R.K., Kohn P.D., Brown T., Egan M.F., Weinberger D.R., Berman K.F. (2005). Regionally Specific Disturbance of Dorsolateral Prefrontal–Hippocampal Functional Connectivity in Schizophrenia. Arch. Gen. Psychiatry.

[bib0096] Micheva, K.D., Wolman, D., Mensh, B.D., Pax, E., Buchanan, J., Smith, S.J., Bock, D.D., 2016. A large fraction of neocortical myelin ensheathes axons of local inhibitory neurons. Elife 5, e15784. 10.7554/elife.15784. PMC4972537 27383052

[bib0097] Miyakawa T., Sumiyoshi S., Deshimaru M., Suzuki T., Tomonari H., Yasuoka F., Tatetsu S. (1972). Electron microscopic study on schizophrenia: Mechanism of pathological changes. Acta Neuropathol.

[bib0098] Miyamae T., Chen K., Lewis D.A., Gonzalez-Burgos G. (2017). Distinct Physiological Maturation of Parvalbumin-Positive Neuron Subtypes in Mouse Prefrontal Cortex. J. Neurosci..

[bib145] Mòdol L., Moissidis M., Marín O. (2023). Somatostatin interneurons control the timing of developmental desynchronization in cortical networks. bioRxiv.

[bib0099] Moghaddam B., Krystal J.H. (2012). Capturing the Angel in “Angel Dust”: Twenty Years of Translational Neuroscience Studies of NMDA Receptor Antagonists in Animals and Humans. Schizophr. Bull..

[bib0100] Mojarad B.A., Engchuan W., Trost B., Backstrom I., Yin Y., Thiruvahindrapuram B., Pallotto L., Mitina A., Khan M., Pellecchia G., Haque B., Guo K., Heung T., Costain G., Scherer S.W., Marshall C.R., Pearson C.E., Bassett A.S., Yuen R.K.C. (2022). Genome-wide tandem repeat expansions contribute to schizophrenia risk. Mol. Psychiatry.

[bib0101] Müller T., Braud S., Jüttner R., Voigt B.C., Paulick K., Sheean M.E., Klisch C., Gueneykaya D., Rathjen F.G., Geiger J.R., Poulet J.F., Birchmeier C. (2018). Neuregulin 3 promotes excitatory synapse formation on hippocampal interneurons. EMBO J.

[bib0102] Murray G.K., Corlett P.R., Clark L., Pessiglione M., Blackwell A.D., Honey G., Jones P.B., Bullmore E.T., Robbins T.W., Fletcher P.C. (2008). Substantia nigra/ventral tegmental reward prediction error disruption in psychosis. Mol. Psychiatry.

[bib0103] Murray R.M., Lewis S.W. (1987). Is schizophrenia a neurodevelopmental disorder?. Br. Med. J. (Clin Res Ed).

[bib0104] Neddens J., Fish K.N., Tricoire L., Vullhorst D., Shamir A., Chung W., Lewis D.A., McBain C.J., Buonanno A. (2011). Conserved Interneuron-Specific ErbB4 Expression in Frontal Cortex of Rodents, Monkeys, and Humans: Implications for Schizophrenia. Biol. Psychiatry.

[bib0105] Ni J., Wunderle T., Lewis C.M., Desimone R., Diester I., Fries P. (2016). Gamma-Rhythmic Gain Modulation. Neuron.

[bib0106] Ni P., Noh H., Park G.-H., Shao Z., Guan Y., Park J.M., Yu S., Park J.S., Coyle J.T., Weinberger D.R., Straub R.E., Cohen B.M., McPhie D.L., Yin C., Huang W., Kim H.-Y., Chung S. (2020). iPSC-derived homogeneous populations of developing schizophrenia cortical interneurons have compromised mitochondrial function. Mol. Psychiatry.

[bib0107] Nowicka D., Soulsby S., Skangiel-Kramska J., Glazewski S. (2009). Parvalbumin-containing neurons, perineuronal nets and experience-dependent plasticity in murine barrel cortex. Eur. J. Neurosci..

[bib0108] Pardiñas A.F., Holmans P., Pocklington A.J., Escott-Price V., Ripke S., Carrera N., Legge S.E., Bishop S., Cameron D., Hamshere M.L., Han J., Hubbard L., Lynham A., Mantripragada K., Rees E., MacCabe J.H., McCarroll S.A., Baune B.T., Breen G., Byrne E.M., Dannlowski U., Eley T.C., Hayward C., Martin N.G., McIntosh A.M., Plomin R., Porteous D.J., Wray N.R., Caballero A., Geschwind D.H., Huckins L.M., Ruderfer D.M., Santiago E., Sklar P., Stahl E.A., Won H., Agerbo E., Als T.D., Andreassen O.A., Bækvad-Hansen M., Mortensen P.B., Pedersen C.B., Børglum A.D., Bybjerg-Grauholm J., Djurovic S., Durmishi N., Pedersen M.G., Golimbet V., Grove J., Hougaard D.M., Mattheisen M., Molden E., Mors O., Nordentoft M., Pejovic-Milovancevic M., Sigurdsson E., Silagadze T., Hansen C.S., Stefansson K., Stefansson H., Steinberg S., Tosato S., Werge T., Consortium G., Consortium C., Collier D.A., Rujescu D., Kirov G., Owen M.J., O'Donovan M.C., Walters J.T.R. (2018). Common schizophrenia alleles are enriched in mutation-intolerant genes and in regions under strong background selection. Nat. Genet..

[bib0109] Pino I.del, García-Frigola C., Dehorter N., Brotons-Mas J.R., Alvarez-Salvado E., Lagrán M.M.de, Ciceri G., Gabaldón M.V., Moratal D., Dierssen M., Canals S., Marin O., Rico B. (2013). Erbb4 Deletion from Fast-Spiking Interneurons Causes Schizophrenia-like Phenotypes. Neuron.

[bib0110] Pocklington A.J., Rees E., Walters J.T.R., Han J., Kavanagh D.H., Chambert K.D., Holmans P., Moran J.L., McCarroll S.A., Kirov G., O'Donovan M.C., Owen M.J. (2015). Novel Findings from CNVs Implicate Inhibitory and Excitatory Signaling Complexes in Schizophrenia. Neuron.

[bib0111] Prabakaran S., Swatton J.E., Ryan M.M., Huffaker S.J., Huang J.-J., Griffin J.L., Wayland M., Freeman T., Dudbridge F., Lilley K.S., Karp N.A., Hester S., Tkachev D., Mimmack M.L., Yolken R.H., Webster M.J., Torrey E.F., Bahn S. (2004). Mitochondrial dysfunction in schizophrenia: evidence for compromised brain metabolism and oxidative stress. Mol. Psychiatry.

[bib0112] Reichelt A.C., Hare D.J., Bussey T.J., Saksida L.M. (2019). Perineuronal Nets: Plasticity, Protection, and Therapeutic Potential. Trends Neurosci.

[bib0113] Reilly T.J., Nottage J.F., Studerus E., Rutigliano G., Micheli A.I.D., Fusar-Poli P., McGuire P. (2018). Gamma band oscillations in the early phase of psychosis: A systematic review. Neurosci. Biobehav. Rev..

[bib0114] Reynolds G.P., Beasley C.L. (2001). GABAergic neuronal subtypes in the human frontal cortex — development and deficits in schizophrenia. J. Chem. Neuroanat..

[bib0115] Rio J.del, Lecea L.de, Ferrer I., Soriano E. (1994). The development of parvalbumin-immunoreactivity in the neocortex of the mouse. Dev. Brain Res..

[bib0116] Ripke S., Neale B.M., Corvin A., Walters J.T.R., Farh K.-H., Holmans P.A., Lee P., Bulik-Sullivan B., Collier D.A., Huang H., Pers T.H., Agartz I., Agerbo E., Albus M., Alexander M., Amin F., Bacanu S.A., Begemann M., Jr R.A.B., Bene J., Bergen S.E., Bevilacqua E., Bigdeli T.B., Black D.W., Bruggeman R., Buccola N.G., Buckner R.L., Byerley W., Cahn W., Cai G., Campion D., Cantor R.M., Carr V.J., Carrera N., Catts S.V., Chambert K.D., Chan R.C.K., Chen R.Y.L., Chen E.Y.H., Cheng W., Cheung E.F.C., Chong S.A., Cloninger C.R., Cohen D., Cohen N., Cormican P., Craddock N., Crowley J.J., Curtis D., Davidson M., Davis K.L., Degenhardt F., Favero J.D., Demontis D., Dikeos D., Dinan T., Djurovic S., Donohoe G., Drapeau E., Duan J., Dudbridge F., Durmishi N., Eichhammer P., Eriksson J., Escott-Price V., Essioux L., Fanous A.H., Farrell M.S., Frank J., Franke L., Freedman R., Freimer N.B., Friedl M., Friedman J.I., Fromer M., Genovese G., Georgieva L., Giegling I., Giusti-Rodríguez P., Godard S., Goldstein J.I., Golimbet V., Gopal S., Gratten J., Haan L.de, Hammer C., Hamshere M.L., Hansen M., Hansen T., Haroutunian V., Hartmann A.M., Henskens F.A., Herms S., Hirschhorn J.N., Hoffmann P., Hofman A., Hollegaard M.V., Hougaard D.M., Ikeda M., Joa I., Julià A., Kahn R.S., Kalaydjieva L., Karachanak-Yankova S., Karjalainen J., Kavanagh D., Keller M.C., Kennedy J.L., Khrunin A., Kim Y., Klovins J., Knowles J.A., Konte B., Kučinskas V., Kucinskiene Z.A., Kuzelova-Ptackova H., Kähler A.K., Laurent C., Keong J.L.C., Lee S.H., Legge S.E., Lerer B., Li M., Li T., Liang K.-Y., Lieberman J., Limborska S., Loughland C.M., Lubinski J., Lönnqvist J., Jr M.M., Magnusson P.K.E., Maher B.S., Maier W., Mallet J., Marsal S., Mattheisen M., Mattingsdal M., McCarley R.W., McDonald C., McIntosh A.M., Meier S., Meijer C.J., Melegh B., Melle I., Mesholam-Gately R.I., Metspalu A., Michie P.T., Milani L., Milanova V., Mokrab Y., Morris D.W., Mors O., Murphy K.C., Murray R.M., Myin-Germeys I., Müller-Myhsok B., Nelis M., Nenadic I., Nertney D.A., Nestadt G., Nicodemus K.K., Nikitina-Zake L., Nisenbaum L., Nordin A., O'Callaghan E., O'Dushlaine C., O'Neill F.A., Oh S.-Y., Olincy A., Olsen L., Os J.van, Consortium P.E.I., Pantelis C., Papadimitriou G.N., Papiol S., Parkhomenko E., Pato M.T., Paunio T., Pejovic-Milovancevic M., Perkins D.O., Pietiläinen O., Pimm J., Pocklington A.J., Powell J., Price A., Pulver A.E., Purcell S.M., Quested D., Rasmussen H.B., Reichenberg A., Reimers M.A., Richards A.L., Roffman J.L., Roussos P., Ruderfer D.M., Salomaa V., Sanders A.R., Schall U., Schubert C.R., Schulze T.G., Schwab S.G., Scolnick E.M., Scott R.J., Seidman L.J., Shi J., Sigurdsson E., Silagadze T., Silverman J.M., Sim K., Slominsky P., Smoller J.W., So H.-C., Spencer C.A., Stahl E.A., Stefansson H., Steinberg S., Stogmann E., Straub R.E., Strengman E., Strohmaier J., Stroup T.S., Subramaniam M., Suvisaari J., Svrakic D.M., Szatkiewicz J.P., Söderman E., Thirumalai S., Toncheva D., Tosato S., Veijola J., Waddington J., Walsh D., Wang D., Wang Q., Webb B.T., Weiser M., Wildenauer D.B., Williams N.M., Williams S., Witt S.H., Wolen A.R., Wong E.H.M., Wormley B.K., Xi H.S., Zai C.C., Zheng X., Zimprich F., Wray N.R., Stefansson K., Visscher P.M., Consortium W.T.C.-C., Adolfsson R., Andreassen O.A., Blackwood D.H.R., Bramon E., Buxbaum J.D., Børglum A.D., Cichon S., Darvasi A., Domenici E., Ehrenreich H., Esko T., Gejman P.V., Gill M., Gurling H., Hultman C.M., Iwata N., Jablensky A.V., Jönsson E.G., Kendler K.S., Kirov G., Knight J., Lencz T., Levinson D.F., Li Q.S., Liu J., Malhotra A.K., McCarroll S.A., McQuillin A., Moran J.L., Mortensen P.B., Mowry B.J., Nöthen M.M., Ophoff R.A., Owen M.J., Palotie A., Pato C.N., Petryshen T.L., Posthuma D., Rietschel M., Riley B.P., Rujescu D., Sham P.C., Sklar P., Clair D.S., Weinberger D.R., Wendland J.R., Werge T., Daly M.J., Sullivan P.F., O'Donovan M.C. (2014). Biological insights from 108 schizophrenia-associated genetic loci. Nature.

[bib0117] Rogers S.L., Rankin-Gee E., Risbud R.M., Porter B.E., Marsh E.D. (2018). Normal Development of the Perineuronal Net in Humans; In Patients with and without Epilepsy. Neuroscience.

[bib0118] Singh T., Poterba T., Curtis D., Akil H., Eissa M.A., Barchas J.D., Bass N., Bigdeli T.B., Breen G., Bromet E.J., Buckley P.F., Bunney W.E., Bybjerg-Grauholm J., Byerley W.F., Chapman S.B., Chen W.J., Churchhouse C., Craddock N., Cusick C.M., DeLisi L., Dodge S., Escamilla M.A., Eskelinen S., Fanous A.H., Faraone S.V., Fiorentino A., Francioli L., Gabriel S.B., Gage D., Taliun S.A.G., Ganna A., Genovese G., Glahn D.C., Grove J., Hall M.-H., Hämäläinen E., Heyne H.O., Holi M., Hougaard D.M., Howrigan D.P., Huang H., Hwu H.-G., Kahn R.S., Kang H.M., Karczewski K.J., Kirov G., Knowles J.A., Lee F.S., Lehrer D.S., Lescai F., Malaspina D., Marder S.R., McCarroll S.A., McIntosh A.M., Medeiros H., Milani L., Morley C.P., Morris D.W., Mortensen P.B., Myers R.M., Nordentoft M., O'Brien N.L., Olivares A.M., Ongur D., Ouwehand W.H., Palmer D.S., Paunio T., Quested D., Rapaport M.H., Rees E., Rollins B., Satterstrom F.K., Schatzberg A., Scolnick E., Scott L.J., Sharp S.I., Sklar P., Smoller J.W., Sobell J.L., Solomonson M., Stahl E.A., Stevens C.R., Suvisaari J., Tiao G., Watson S.J., Watts N.A., Blackwood D.H., Børglum A.D., Cohen B.M., Corvin A.P., Esko T., Freimer N.B., Glatt S.J., Hultman C.M., McQuillin A., Palotie A., Pato C.N., Pato M.T., Pulver A.E., Clair D.St., Tsuang M.T., Vawter M.P., Walters J.T., Werge T.M., Ophoff R.A., Sullivan P.F., Owen M.J., Boehnke M., O'Donovan M.C., Neale B.M., Daly M.J. (2022). Rare coding variants in ten genes confer substantial risk for schizophrenia. Nature.

[bib0119] Sohal V.S., Zhang F., Yizhar O., Deisseroth K. (2009). Parvalbumin neurons and gamma rhythms enhance cortical circuit performance. Nature.

[bib0120] Somogyi P., Tamás G., Lujan R., Buhl E.H. (1998). Salient features of synaptic organisation in the cerebral cortex. Brain Res. Rev..

[bib0121] Sørensen H.J., Mortensen E.L., Schiffman J., Reinisch J.M., Maeda J., Mednick S.A. (2010). Early developmental milestones and risk of schizophrenia: A 45-year follow-up of the Copenhagen Perinatal Cohort. Schizophr. Res..

[bib0122] Stedehouder J., Couey J.J., Brizee D., Hosseini B., Slotman J.A., Dirven C.M.F., Shpak G., Houtsmuller A.B., Kushner S.A. (2017). Fast-spiking Parvalbumin Interneurons are Frequently Myelinated in the Cerebral Cortex of Mice and Humans. Cereb. Cortex.

[bib0123] Steullet P., Cabungcal J.-H., Coyle J., Didriksen M., Gill K., Grace A.A., Hensch T.K., LaMantia A.-S., Lindemann L., Maynard T.M., Meyer U., Morishita H., O'Donnell P., Puhl M., Cuénod M., Do K.Q. (2017). Oxidative stress-driven parvalbumin interneuron impairment as a common mechanism in models of schizophrenia. Mol. Psychiatry.

[bib0124] Stilo S.A., Murray R.M. (2010). The epidemology of schizophrenia: replacing dogma with knowledge. Dialogues Clin. Neurosci..

[bib0125] Szegedi V., Paizs M., Baka J., Barzó P., Molnár G., Tamas G., Lamsa K. (2020). Robust perisomatic GABAergic self-innervation inhibits basket cells in the human and mouse supragranular neocortex. eLife.

[bib0126] Takács V.T., Szőnyi A., Freund T.F., Nyiri G., Gulyás A.I. (2015). Quantitative ultrastructural analysis of basket and axo-axonic cell terminals in the mouse hippocampus. Brain Struct. Funct..

[bib0127] Thuné H., Recasens M., Uhlhaas P.J. (2016). The 40-Hz Auditory Steady-State Response in Patients With Schizophrenia: A Meta-analysis. JAMA Psychiatry.

[bib0128] Tikka S.K., Yadav S., Nizamie S.H., Das B., Tikka D.L., Goyal N. (2013). Schneiderian First Rank Symptoms and Gamma Oscillatory Activity in Neuroleptic Naïve First Episode Schizophrenia: A 192 Channel EEG Study. Psychiatry Investig.

[bib0129] Ting A.K., Chen Y., Yin D.-M., Shen C., Tao Y., Xiong W.-C., Mei L. (2011). Neuregulin 1 promotes excitatory synapse development and function in GABAergic interneurons. J. Neurosci..

[bib0130] Trubetskoy V, Pardiñas AF, Qi T, Panagiotaropoulou G, Awasthi S, Bigdeli TB, Bryois J, Chen C-Y, Dennison CA, Hall LS, Lam M, Watanabe K, Frei O, Ge T, Harwood JC, Koopmans F, Magnusson S, Richards AL, Sidorenko J, Wu Y, Zeng J, Grove J, Kim M, Li Z, Voloudakis G, Zhang W, Adams M, Agartz I, Atkinson EG, Agerbo E, Eissa MA, Albus M, Alexander M, Alizadeh BZ, Alptekin K, Als TD, Amin F, Arolt V, Arrojo M, Athanasiu L, Azevedo MH, Bacanu SA, Bass NJ, Begemann M, Belliveau RA, Bene J, Benyamin B, Bergen SE, Blasi G, Bobes J, Bonassi S, Braun A, Bressan RA, Bromet EJ, Bruggeman R, Buckley PF, Buckner RL, Bybjerg-Grauholm J, Cahn W, Cairns MJ, Calkins ME, Carr VJ, Castle D, Catts SV, Chambert KD, Chan RCK, Chaumette B, Cheng W, Cheung EFC, Chong SA, Cohen D, Consoli A, Cordeiro Q, Costas J, Curtis C, Davidson M, Davis KL, Haan L de, Degenhardt F, DeLisi LE, Demontis D, Dickerson F, Dikeos D, Dinan T, Djurovic S, Duan J, Ducci G, Dudbridge F, Eriksson JG, Fañanás L, Faraone SV, Fiorentino A, Forstner A, Frank J, Freimer NB, Fromer M, Frustaci A, Gadelha A, Genovese G, Gershon ES, Giannitelli M, Giegling I, Giusti-Rodríguez P, Godard S, Goldstein JI, Peñas JG, González-Pinto A, Gopal S, Gratten J, Green MF, Greenwood TA, Guillin O, Gülöksüz S, Gur RE, Gur RC, Gutiérrez B, Hahn E, Hakonarson H, Haroutunian V, Hartmann AM, Harvey C, Hayward C, Henskens FA, Herms S, Hoffmann P, Howrigan DP, Ikeda M, Iyegbe C, Joa I, Julià A, Kähler AK, Kam-Thong T, Kamatani Y, Karachanak-Yankova S, Kebir O, Keller MC, Kelly BJ, Khrunin A, Kim S-W, Klovins J, Kondratiev N, Konte B, Kraft J, Kubo M, Kučinskas V, Kučinskiene ZA, Kusumawardhani A, Kuzelova-Ptackova H, Landi S, Lazzeroni LC, Lee PH, Legge SE, Lehrer DS, Lencer R, Lerer B, Li Miaoxin, Lieberman J, Light GA, Limborska S, Liu C-M, Lönnqvist J, Loughland CM, Lubinski J, Luykx JJ, Lynham A, Macek M, Mackinnon A, Magnusson PKE, Maher BS, Maier W, Malaspina D, Mallet J, Marder SR, Marsal S, Martin AR, Martorell L, Mattheisen M, McCarley RW, McDonald C, McGrath JJ, Medeiros H, Meier S, Melegh B, Melle I, Mesholam-Gately RI, Metspalu A, Michie PT, Milani L, Milanova V, Mitjans M, Molden E, Molina E, Molto MD, Mondelli V, Moreno C, Morley CP, Muntané G, Murphy KC, Myin-Germeys I, Nenadić I, Nestadt G, Nikitina-Zake L, Noto C, Nuechterlein KH, O'Brien NL, O'Neill FA, Oh S-Y, Olincy A, Ota VK, Pantelis C, Papadimitriou GN, Parellada M, Paunio T, Pellegrino R, Periyasamy S, Perkins DO, Pfuhlmann B, Pietiläinen O, Pimm J, Porteous D, Powell J, Quattrone D, Quested D, Radant AD, Rampino A, Rapaport MH, Rautanen A, Reichenberg A, Roe C, Roffman JL, Roth J, Rothermundt M, Rutten BPF, Saker-Delye S, Salomaa V, Sanjuan J, Santoro ML, Savitz A, Schall U, Scott RJ, Seidman LJ, Sharp SI, Shi J, Siever LJ, Sigurdsson E, Sim K, Skarabis N, Slominsky P, So H-C, Sobell JL, Söderman E, Stain HJ, Steen NE, Steixner-Kumar AA, Stögmann E, Stone WS, Straub RE, Streit F, Strengman E, Stroup TS, Subramaniam M, Sugar CA, Suvisaari J, Svrakic DM, Swerdlow NR, Szatkiewicz JP, Ta TMT, Takahashi A, Terao C, Thibaut F, Toncheva D, Tooney PA, Torretta S, Tosato S, Tura GB, Turetsky BI, Üçok A, Vaaler A, Amelsvoort T van, Winkel R van, Veijola J, Waddington J, Walter H, Waterreus A, Webb BT, Weiser M, Williams NM, Witt SH, Wormley BK, Wu JQ, Xu Z, Yolken R, Zai CC, Zhou W, Zhu F, Zimprich F, Atbaşoğlu EC, Ayub M, Benner C, Bertolino A, Black DW, Bray NJ, Breen G, Buccola NG, Byerley WF, Chen WJ, Cloninger CR, Crespo-Facorro B, Donohoe G, Freedman R, Galletly C, Gandal MJ, Gennarelli M, Hougaard DM, Hwu H-G, Jablensky AV, McCarroll SA, Moran JL, Mors O, Mortensen PB, Müller-Myhsok B, Neil AL, Nordentoft M, Pato MT, Petryshen TL, Pirinen M, Pulver AE, Schulze TG, Silverman JM, Smoller JW, Stahl EA, Tsuang DW, Vilella E, Wang S-H, Xu S, Dai N, Wenwen Q, Wildenauer DB, Agiananda F, Amir N, Antoni R, Arsianti T, Asmarahadi A, Diatri H, Djatmiko P, Irmansyah I, Khalimah S, Kusumadewi I, Kusumaningrum P, Lukman PR, Nasrun MW, Safyuni NS, Prasetyawan P, Semen G, Siste K, Tobing H, Widiasih N, Wiguna T, Wulandari D, Evalina N, Hananto AJ, Ismoyo JH, Marini TM, Henuhili S, Reza M, Yusnadewi S, Abyzov A, Akbarian S, Ashley-Koch A, Bakel H van, Breen M, Brown M, Bryois J, Carlyle B, Charney A, Coetzee G, Crawford G, Dracheva S, Emani P, Farnham P, Fromer M, Galeev T, Gandal M, Gerstein M, Giase G, Girdhar K, Goes F, Grennan K, Gu M, Guerra B, Gursoy G, Hoffman G, Hyde T, Jaffe A, Jiang S, Jiang Y, Kefi A, Kim Y, Kitchen R, Knowles JA, Lay F, Lee D, Li Mingfeng, Liu C, Liu S, Mattei E, Navarro F, Pan X, Peters MA, Pinto D, Pochareddy S, Polioudakis D, Purcaro M, Purcell S, Pratt H, Reddy T, Rhie S, Roussos Panagiotis, Rozowsky J, Sanders S, Sestan N, Sethi A, Shi X, Shieh A, Swarup V, Szekely A, Wang D, Warrell J, Weissman S, Weng Z, White K, Wiseman J, Witt H, Won H, Wood S, Wu F, Xu X, Yao L, Zandi P, Arranz MJ, Bakker S, Bender S, Bramon E, Collier DA, Crepo-Facorro B, Hall J, Iyegbe C, Kahn R, Lawrie S, Lewis C, Lin K, Linszen DH, Mata I, McIntosh A, Murray RM, Ophoff RA, Os J van, Powell J, Rujescu D, Walshe M, Weisbrod M, Achsel T, Andres-Alonso M, Bagni C, Bayés À, Biederer T, Brose N, Brown TC, Chua JJE, Coba MP, Cornelisse LN, Jong APH de, Juan-Sanz J de, Dieterich DC, Feng G, Goldschmidt HL, Gundelfinger ED, Hoogenraad C, Huganir RL, Hyman SE, Imig C, Jahn R, Jung H, Kaeser PS, Kim E, Koopmans F, Kreutz MR, Lipstein N, MacGillavry HD, Malenka R, McPherson PS, O'Connor V, Pielot R, Ryan TA, Sahasrabudhe D, Sala C, Sheng M, Smalla K-H, Smit AB, Südhof TC, Thomas PD, Toonen RF, Weering JRT van, Verhage M, Verpelli C, Adolfsson R, Arango C, Baune BT, Belangero SI, Børglum AD, Braff D, Bramon E, Buxbaum JD, Campion D, Cervilla JA, Cichon S, Collier DA, Corvin A, Curtis D, Forti MD, Domenici E, Ehrenreich H, Escott-Price V, Esko T, Fanous AH, Gareeva A, Gawlik M, Gejman PV, Gill M, Glatt SJ, Golimbet V, Hong KS, Hultman CM, Hyman SE, Iwata N, Jönsson EG, Kahn RS, Kennedy JL, Khusnutdinova E, Kirov G, Knowles JA, Krebs M-O, Laurent-Levinson C, Lee J, Lencz T, Levinson DF, Li QS, Liu J, Malhotra AK, Malhotra D, McIntosh A, McQuillin A, Menezes PR, Morgan VA, Morris DW, Mowry BJ, Murray RM, Nimgaonkar V, Nöthen MM, Ophoff RA, Paciga SA, Palotie A, Pato CN, Qin S, Rietschel M, Riley BP, Rivera M, Rujescu D, Saka MC, Sanders AR, Schwab SG, Serretti A, Sham PC, Shi Y, Clair DS, Stefánsson H, Stefansson K, Tsuang MT, Os J van, Vawter MP, Weinberger DR, Werge T, Wildenauer Dieter B, Yu X, Yue W, Holmans PA, Pocklington AJ, Roussos Panos, Vassos E, Verhage M, Visscher PM, Yang J, Posthuma D, Andreassen OA, Kendler KS, Owen MJ, Wray NR, Daly MJ, Huang H, Neale BM, Sullivan PF, Ripke S, Walters JTR, O'Donovan MC, Haan L de, Amelsvoort T van, Winkel R van, Gareeva A, Sham PC, Shi Y, Clair DS, Os J van (2022). Mapping genomic loci implicates genes and synaptic biology in schizophrenia. Nature.

[bib0131] Uhlhaas P.J., Singer W. (2012). Neuronal dynamics and neuropsychiatric disorders: toward a translational paradigm for dysfunctional large-scale networks. Neuron.

[bib0132] Uhlhaas P.J., Singer W. (2010). Abnormal neural oscillations and synchrony in schizophrenia. Nat. Rev. Neurosci..

[bib0133] Uranova N.A., Vikhreva O.V., Rachmanova V.I., Orlovskaya D.D. (2011). Ultrastructural Alterations of Myelinated Fibers and Oligodendrocytes in the Prefrontal Cortex in Schizophrenia: A Postmortem Morphometric Study. Schizophr. Res. Treat..

[bib0134] Vierling-Claassen D., Siekmeier P., Stufflebeam S., Kopell N. (2008). Modeling GABA alterations in schizophrenia: a link between impaired inhibition and altered gamma and beta range auditory entrainment. J. Neurophysiol..

[bib0135] Walsh T., McClellan J.M., McCarthy S.E., Addington A.M., Pierce S.B., Cooper G.M., Nord A.S., Kusenda M., Malhotra D., Bhandari A., Stray S.M., Rippey C.F., Roccanova P., Makarov V., Lakshmi B., Findling R.L., Sikich L., Stromberg T., Merriman B., Gogtay N., Butler P., Eckstrand K., Noory L., Gochman P., Long R., Chen Z., Davis S., Baker C., Eichler E.E., Meltzer P.S., Nelson S.F., Singleton A.B., Lee M.K., Rapoport J.L., King M.-C., Sebat J. (2008). Rare structural variants disrupt multiple genes in neurodevelopmental pathways in schizophrenia. Science.

[bib0136] Wang H., Gao W. (2009). Cell Type-Specific Development of NMDA Receptors in the Interneurons of Rat Prefrontal Cortex. Neuropsychopharmacology.

[bib0137] Wang H., Liu F., Chen W., Sun X., Cui W., Dong Z., Zhao K., Zhang H., Li H., Xing G., Fei E., Pan B.-X., Li B.-M., Xiong W.-C., Mei L. (2018). Genetic recovery of ErbB4 in adulthood partially restores brain functions in null mice. Proc. Natl. Acad. Sci..

[bib0138] Wang H.-X., Gao W.-J. (2010). Development of calcium-permeable AMPA receptors and their correlation with NMDA receptors in fast-spiking interneurons of rat prefrontal cortex. J. Physiol. (Lond).

[bib0139] Weinberger D.R. (1987). Implications of Normal Brain Development for the Pathogenesis of Schizophrenia. Arch Gen Psychiatry.

[bib0140] Weinberger D.R., Berman K.F., Zec R.F. (1986). Physiologic Dysfunction of Dorsolateral Prefrontal Cortex in Schizophrenia: I. Regional Cerebral Blood Flow Evidence. Arch. Gen. Psychiatry.

[bib0141] Yadav S., Nizamie S.H., Das B., Das J., Tikka S.K. (2021). Resting state quantitative electroencephalogram gamma power spectra in patients with first episode psychosis: An observational study. Asian J. Psychiatry.

[bib0142] Ye Q., Miao Q. (2013). Experience-dependent development of perineuronal nets and chondroitin sulfate proteoglycan receptors in mouse visual cortex. Matrix Biol.

[bib0143] Yin D.-M., Sun X.-D., Bean J.C., Lin T.W., Sathyamurthy A., Xiong W.-C., Gao T.-M., Chen Y.-J., Mei L. (2013). Regulation of spine formation by ErbB4 in PV-positive interneurons. J. Neurosci..

[bib0144] Zhang Z.J., Reynolds G.P. (2002). A selective decrease in the relative density of parvalbumin-immunoreactive neurons in the hippocampus in schizophrenia. Schizophr. Res..

